# High Throughput scRNA-Seq Provides Insights Into Leydig Cell Senescence Induced by Experimental Autoimmune Orchitis: A Prominent Role of Interstitial Fibrosis and Complement Activation

**DOI:** 10.3389/fimmu.2021.771373

**Published:** 2022-01-17

**Authors:** Yinchuan Li, Panpan Mi, Jiabao Wu, Yunge Tang, Xiaohua Liu, Jinmei Cheng, Yingying Huang, Weibing Qin, C. Yan Cheng, Fei Sun

**Affiliations:** ^1^ Institute of Reproductive Medicine, Medical School of Nantong University, Nantong, China; ^2^ NHC Key Laboratory of Male Reproduction and Genetics, Guangdong Provincial Reproductive Science Institute (Guangdong Provincial Fertility Hospital), Guangzhou, China; ^3^ The Mary M. Wohlford Laboratory for Male Contraceptive Research, Center for Biomedical Research, Population Council, New York, NY, United States

**Keywords:** scRNA-Seq, Leydig cells, androgen synthesis, cytokines, experimental autoimmune orchitis (EAO), glutathione metabolism, fibrosis, complement

## Abstract

Leydig cells (Lc), located in the interstitial space of the testis between seminiferous tubules, produce 95% of testosterone in male individuals, which is pivotal for male sexual differentiation, spermatogenesis, and maintenance of the male secondary sex characteristics. Lc are prone to senescence in aging testes, resulting in compromised androgen synthesis capability upon aging. However, little is known about whether Lc undergo senescence in a chronic inflammatory environment. To investigate this question, mouse models of experimental autoimmune orchitis (EAO) were used, and Lc were analyzed by high throughput scRNA-Seq. Data were screened and analyzed by correlating signaling pathways with senescence, apoptosis, androgen synthesis, and cytokine/chemokine signaling pathways. EAO did induce Lc senescence, and Lc senescence in turn antagonized androgen synthesis. Based on the correlation screening of pathways inducing Lc senescence, a plethora of pathways were found to play potential roles in triggering Lc senescence during EAO, among which the *Arf6* and angiopoietin receptor pathways were highly correlated with senescence signature. Notably, complement and interstitial fibrosis activated by EAO worsened Lc senescence and strongly antagonized androgen synthesis. Furthermore, most proinflammatory cytokines enhanced both senescence and apoptosis in Lc and spermatogonia (Sg) during EAO, and proinflammatory cytokine antagonism of the glutathione metabolism pathway may be key in inducing cellular senescence during EAO.

## Introduction

Lc senescence in aging testes leads to reduced serum testosterone levels and hypogonadism, affecting male fertility in 2%–3% of aging men (1). The prevalent viewpoint is that the factors contributing to Lc senescence during aging are intrinsic factors, such as the reduced steroidogenic capacity, lowered response to luteinizing hormone (LH) stimulation, reduced cholesterol import, reduced cholesterol synthesis, and compromised transport to mitochondria in aging Lc (2, 3). Reduced Lc antioxidant capacity is the major cause in this process (3–10). The age-related decline of Lc function also involves extrinsic factors such as chronic inflammation (11), which has been largely neglected in earlier studies. For example, Lc are intimately connected functionally to various immune cells in the interstitium (12). Local inflammation in the testes also activates macrophages at the site to produce reactive oxygen species (ROS) such as hydrogen peroxide, which can perturb Lc function in the interstitium (13). Low serum androgen levels in aged men usually correlates with elevated levels of circulating proinflammatory cytokines (14, 15).

Accumulating evidence indicates that low-grade chronic systemic inflammation established during physiological aging known as inflammaging affects nearly all tissues in organs during aging (16, 17). Inflammaging leads to over-abundance of ROS, thereby enhancing cellular oxidation that damages multiple cellular components (18). Notably, inflammaging is usually accompanied by changes in the secretion of proinflammatory cytokines [e.g., increased interleukin (IL)-6, tumor necrosis factor (TNF)-α], growth factors, matrix metalloproteinases, and other molecules (e.g., increased acute-phase reactants such as C-reactive protein, reduced IL-10). These changes are collectively called the senescence-associated secretory phenotype (SASP), which further deteriorates the local microenvironment by impairing the maintenance of immunological homeostasis (19). Therefore, the oxidation-inflammation theory (oxi-inflamm-aging) is proposed to be the main reason of aging (20).

Experimental autoimmune orchitis (EAO) is a widely used model for chronic testicular inflammation and autoimmunity that offers an *in vivo* tool to investigate the pathological details of inflammaging in testis. EAO is accompanied by infiltration of the interstitium by immune cells (including macrophages, dendritic cells, neutrophils, mast cells, B cells, T cells, and NK cells), generation of autoantibodies against testicular antigens, production of proinflammatory mediators (e.g., NO, CCL2, TNF-α, IFN-γ, IL-6, IL-12, IL-17, IL-23) or activins, and dysregulation of steroidogenesis with reduced levels of serum testosterone (21–25). Studies have shown that single-cell RNA sequencing (scRNA-Seq) is a robust tool to monitor changes in testicular cell populations, including Lc, and their mutual communication pathways in cell senescence and inflammation (26, 27).

In this study, we utilize the mouse EAO model to monitor the inflammaging process in the testes through scRNA-Seq-based transcriptome profiling. The primary goal was to screen senescence- and androgen-related signaling and inflammatory response pathways to dissect the triple interplay among them in Lc with the aim of providing deep insights into the impact of proinflammatory cytokines on androgen synthesis and senescence in normal adult Lc and orchitic Lc.

## Materials and Methods

### Mice and EAO Models

We used 10–12-week-old male C57BL/6N mice for this study. All animal protocols and experiments were approved by the University of Nantong Animal Care and Use Committee and the Animal Care and Use Office. Animals were housed in 12-h light/12-h dark cycles, and food and water were freely accessible. Induction of EAO was conducted according to an earlier report with minor modifications (28). Mice testicular homogenate was prepared from decapsulated testes and homogenized in sterile 1× phosphate-buffered saline (PBS) at a ratio of 1:1. Then, the homogenates were centrifuged at 1,000 rpm for 5 min. The supernatant was mixed either with complete Freund’s adjuvant (the first immunization) or incomplete Freund’s adjuvant (the last two immunizations) at a ratio of 1:1. Animals were immunized three times at 14-day intervals, and each time was followed by i.p. injection of 100 ng *Bordetella pertussis* toxin (Calbiochem, Darmstadt, Germany) in 100 μl 1× PBS. Each animal was injected s.c. dorsally on four sites with a total volume of 200 μl. The normal control group was named N, and the testicular cells digested with trypsin/ethylenediaminetetraacetic acid (EDTA) named Nt and others digested with collagenase named Nc as per earlier report (29). The orchitic groups were designated O30 (30 days after the first immunization) and O50 (50 days after the first immunization). The testicular cells of O50 were digested with trypsin/EDTA and named O50t.

### Single-Cell Suspensions, Cell Capture, and Library Preparation

Three unilateral decapsulated testes from three mice collected either from group O50 or N were pooled. The cell suspension preparation, cell capture, and library preparation were conducted according to earlier reports (29, 30). scRNA-seq libraries were prepared following the protocol of the GEXSCOPE™ Single-Cell RNA Library Kit (Singleron Biotechnologies, China). Libraries were sequenced on the Illumina HiSeq X using 150 bp paired-end reads.

### scRNA-Seq Data Analysis

The raw data analysis pipeline was conducted essentially according to our previous report (29). Cells were integrated and filtered under Seurat 3.2.2 (nFeature_RNA > 200, nFeature_RNA < 7,000, percent.mt < 20 and min.cells = 3) (31). Batch effects were removed by sctransform 0.3.1 in Seurat. All cell clusters except Sd6, which was merged manually, were divided in an unbiased manner. In order to identify the differentially expressed genes, the function FindMarkers of Seurat was used (test.use = “wilcox”, logfc.threshold = 0.25, min.pct = 0.2). Up- and downregulated genes were filtered (p_val ≤ 0.05) as differentially expressed genes between O50t and Nt. The gene set enrichment was performed following the competitive gene set enrichment test CAMERA embedded in the SingleSeqGset (version 0.1.2.9000) R package (32). GO enrichment analysis between O50t and Nt was also obtained based on the gene set enrichment analysis [gene set enrichment analysis (GSEA) 4.0.3] against C5 (CC), C2 [Kyoto Encyclopedia of Genes and Genomes (KEGG), Pathway Interaction Database (PID)], and C1 (Hallmark). A total of 1,428 transcription factors were annotated in the data according to the gene symbol of 1,721 mouse transcription factors collected in cisTarget databases. Due to the very high expression level of Hmgb4, Spz1, and Tfam in elongating spermatids by delayed transcription, the three transcription factors were opted out in the correlation analysis because of their high expression background in other cell clusters. The average expression level of transcription factors was set to be no <0.5. For correlation analysis, gene sets, pathways, or signatures were calculated on the percentage of all counts that belong to each set of features *via* the PercentageFeatureSet function in Seurat package. Then, Pearson correlations were calculated among these gene sets, pathways, or signatures. Gene correlation analysis was performed directly on the data matrix by Pearson correlation method. The top 20 TFs that positively or negatively correlated with target genes were selected according to the ranks of correlation coefficient r-value.

Receptor–ligand pairs were generated by Linux package CellPhoneDB (33). Cell communication networks were also generated by R package CellChat (34).

Five signatures were used for correlation analysis: Senescence_signature, Androgen_synthesis_signature, mCRPs, Complement, and Collagen. Genes included in the signatures are listed in **Supplementary Table S1**.

### Immunofluorescence, Senescence-Associated β-Galactosidase Activity, and Masson’s Trichrome Staining

Formalin-fixed paraffin-embedded mouse testis sections of 5 µm thickness were used for immunofluorescence (IF) staining. Sections were deparaffinized and antigens retrieved by heating in sodium citrate buffer. Primary autoantibodies were derived from the serum of O50 and adult normal control mice. Secondary antibody used was Alexa Fluor 488-conjugated anti-mouse (Thermo Fisher). Testis cross-sections on microscopic slides were then counterstained with Hoechst 33342 and observed under a fluorescent microscope (Axio Imager M2, Zeiss, Germany). β-Galactosidase activity, which is used as a biomarker of senescence, was detected using a staining kit (C0602) from Beyotime Technology (Shanghai, China) according to the manufacturer’s instructions. Collagen fibers were visualized with Masson’s trichrome stain using a kit (XY1427N) from Shanghai Xin Yu Biotechnology (China) according to the manufacturer’s instructions.

## Results

### Chronic Orchitis Induces Lc Senescence

**Figure 1:**
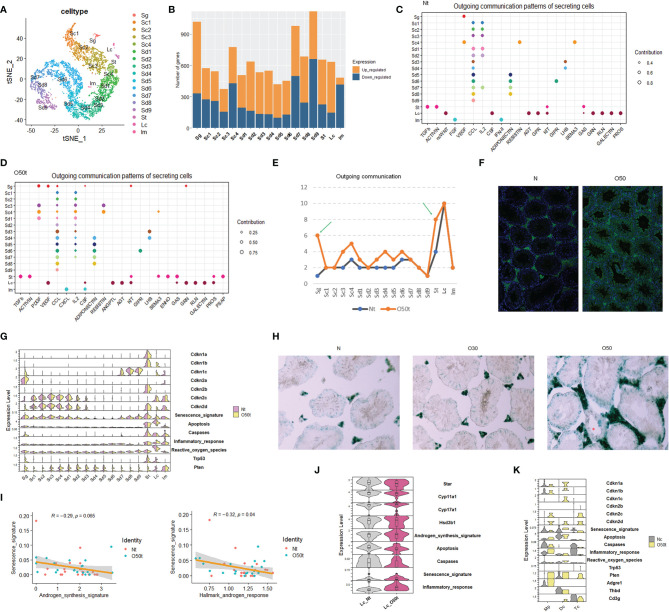
scRNA-Seq of testes from orchitic B6 mice (O50) and normal adult mice (N). **(A)** Dimensionality reduction of O50t and Nt cells with t-Distributed Stochastic Neighbor Embedding (tSNE). Nt, trypsin/EDTA-digested normal adult mice testes; O50t, trypsin/EDTA-digested O50 mice testes (50 days after the first immunization); Sg, spermatogonia; Sc, spermatocytes; Sd, spermatid; St, Sertoli cells; Im, mixture of immune cells. **(B)** Counts of differentially expressed genes between O50t and Nt in each cell cluster. **(C, D)** The cell outgoing communication patterns in Nt and O50t, respectively, detected by CellChat with default settings. **(E)** Counts of the number of outgoing communication patterns in Nt and O50t. **(F)** IF staining of the normal mouse testis slices with the auto-antibodies from the serum of O50 mice and the control serum of N mice. **(G)** The overall transcriptomic profiles of genes, signatures, and pathways from senescence, Hallmark apoptosis, Hallmark inflammatory response, and enzymes of reactive oxygen species (Hallmark) across all the 17 cell clusters between Nt and O50t. **(H)** β-Galactosidase activity staining of N, O30, and O50 testes slices. **(I)** Correlational analyses of senescence signature with androgen synthesis signature or with androgen response pathway in Leydig cells. **(J)** Violin plot showing the relative expression level of genes and signatures of senescence, androgen synthesis, Hallmark apoptosis, caspases, and Hallmark inflammatory responses between Lc_Nt and Lc_O50t. **(K)** The expression pattern of genes, signatures, and pathways from senescence, Hallmark apoptosis, Hallmark inflammatory response, and enzymes of Hallmark reactive oxygen species in Mp (macrophages), Dc (dendritic cells), and Tc (T cells) from Nc and O50t samples. Nc, collagenase digested normal adult mouse testis samples.

Using our scRNA-Seq datasets, Nt and O50t were integrated to remove batch effects; then, 6,785 clean cells were obtained and divided into 17 cell clusters, including 1 cluster of Sg, 4 clusters of spermatocytes (Sc), 9 clusters of spermatids (Sd), 1 cluster of Lc, 1 cluster of Sertoli cells (St), and 1 mixture of immune cells (Im) (**Figure 1A**). The top algorithmic cell markers are shown in **Supplementary Figure S1A**. Orchitis led to extensive changes in gene expression (**Figure 1B**), especially in Sg and Sd7-Sd9 spermatids, which were located either adjacent to the interstitium or faced the lumens of seminiferous tubules, respectively, making these cells more vulnerable to be affected by immune factors. In parallel to the gene expression changes, the tissue morphology of O50 also changed with thickened tunica albuginea (**Supplementary Figure S1B**). Inflammation also remarkably altered the outgoing communication patterns by which the sender cells coordinate with other cells (**Figures 1C, D** **)**, especially for Sg and St cells (**Figure 1E**). Orchitis was accompanied by increased infiltration of antibodies against germ cells (**Figure 1F**, **Supplementary Figure S1C**). Orchitis also altered the expression of key genes from senescence and apoptosis pathways in some cell clusters, such as an increase in senescence markers *Cdkn1b* and *Cdkn1c* in Lc cells and an increase in apoptosis markers *Trp53/p53* and *Pten* in Sg cells (**Figure 1G**). To verify the senescence of Lc cells, β-galactosidase activity was stained, and indeed, the interstitial cells in O50t sample had higher β-galactosidase activity (**Figure 1H**).

We next assessed the correlation between senescence signature with the androgen synthesis signature and Hallmark androgen response pathway in Lc. The senescence signature was significantly negatively correlated with the Hallmark androgen response pathway and trended towards a significant negative correlation with the androgen synthesis signature (**Figure 1I**). Star and Cyp11a1, the two key enzymes in the androgen synthesis pathway, were downregulated, and the senescence signature was upregulated in Lc_O50t (**Figure 1J**). In addition to Lc, macrophages and other minor cell populations from the interstitium of testis also became potential candidates of senescent cells. To investigate this, instead of Nt, the scRNA-Seq data of the Nc sample and O50t sample were integrated to enrich more Im, which were subdivided into three distinct cell clusters: Mp (macrophages), Dc (dendritic cells), and Tc (T cells). By comparing the genes and signatures of senescence and apoptosis, we found that Dc cells, but not Mp or Tc, had a higher potential to commit senescence.

### Downregulation of Redox-Related Pathways by EAO and the Screening of Senescence-Related Pathways in Lc

**Figure 2:**
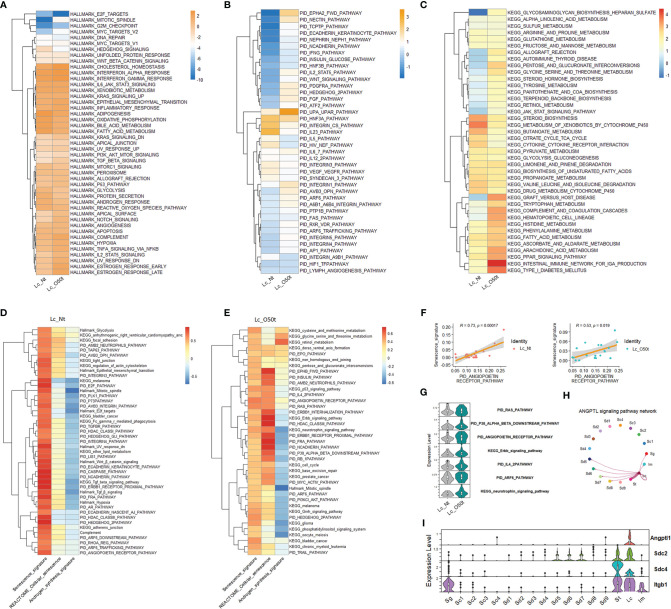
Screening of senescence-correlated pathways in Leydig cells during EAO. **(A–C)** The expression levels of Hallmark gene sets, the top 40 KEGG pathways in Lc_O50t, and the top 40 PID pathways in Lc_O50t. **(D)** The top correlation ranks (R ≥ 0.6) of senescence signature with Hallmark gene sets, KEGG pathways, and PID pathways in Lc_Nt. **(E)** The top correlation ranks (R ≥ 0.32) of senescence signature with Hallmark gene sets, KEGG pathways, and PID pathways in Lc_O50t. **(F)** The correlation of senescence signature with PID_ANGIOPOIETIN_RECEPTOR_PATHWAY in Lc_Nt and Lc_O50t. **(G)** The expression level of several pathways positively correlated with Lc senescence during orchitis. **(H)** The ANGPTL signaling pathway networks among testis cell clusters. **(I)** The expression profiles of four highly expressed members from ANGPTL signaling pathway in mouse testes.

To elucidate the regulation of Lc senescence and androgen synthesis during orchitis, we compared the relative expression levels of Hallmark gene sets, KEGG pathways and PID signaling pathways between Lc_Nt and Lc_O50t (**Figures 2A–C**). As expected, orchitis potentially elevated the inflammatory response in Lc. For example, the levels of gene sets and signatures of Hallmark complement, Hallmark Il2_Stat5, Hallmark Tnf-*α* signaling *via* Nfkb, Hallmark TGF-β signaling, KEGG arachidonic acid metabolism and KEGG complement,a and coagulation cascades were increased during EAO. In addition, the redox status and androgen synthesis pathways were potentially compromised during EAO. For example, the levels of gene sets of Hallmark oxidative phosphorylation, Hallmark reactive oxygen species, Hallmark fatty acid metabolism, Hallmark androgen response, Hallmark glycolysis, and KEGG steroid biosynthesis were considerably downregulated. More importantly, elevation of the Hallmark p53 pathway and PID Arf6 pathway implied that senescence may occur in Lc during EAO (**Figures 2A, B** **)**, as both pathways are usually regarded to be involved in senescence (35).

Next, we screened the contributing pathways to Lc senescence *via* correlation analysis. To accomplish this, the feasible collection of a senescence signature was the key because senescence-related genes were normally expressed in most cells such as the collection of REACTOME cellular senescence pathway. Thus, REACTOME cellular senescence pathway was not ideal for identifying the senescence signature under normal conditions. In this study, we used the classic signatures of senescence: Cdkn1a, Cdkn1b, Cdkn1c, Cdkn2a, Cdkn2b, Cdkn2c, and Cdkn2d. As expected, the highly correlated pathways with senescence signature were different to that of REACTOME cellular senescence pathway in Lc_Nt (**Figure 2D**). But alternatively, both shared many positively correlated pathways in Lc_O50t (**Figure 2E**), suggesting that senescence occurred in Lc_O50t. Notably, the androgen synthesis signature displayed opposite correlation patterns with senescence whether in Lc_Nt or Lc_O50t (**Figures 2D, E** **)**. More interestingly, the PID_RAS, PID P38-alpha-beta downstream, PID angiopoietin receptor, PID IL4-2, PID ARF6, PID FRA, PID PI3KCL-AKT, KEGG neurotrophin signaling, KEGG p53, KEGG Erbb signaling, and KEGG cell cycle pathways all displayed a highly positive correlation with senescence signature. The PID angiopoietin receptor pathway displayed the highest correlation coefficient, suggesting a high potential for inducing Lc senescence during orchitis (**Figures 2E, F** **)**. Many of these signaling pathways, including the PID Arf6 and PID angiopoietin receptor pathways, were increased in Lc during orchitis (**Figure 2G**). The angiopoietin receptor pathway network mainly existed among Lc, St, and Sd with unknown roles (**Figures 2H, I** **)**.

Taken collectively, the relative expression levels of the Hallmark gene sets, PID signaling pathways (top 40), and KEGG signaling pathways (top 40) helped identify enriched signaling pathways in Lc during EAO. The correlation analysis of all three databases of signaling pathways with a senescence signature facilitated screening the closely correlated signaling pathways with senescence during EAO.

### The Impact of Orchitis on Androgen Synthesis-Correlated Pathways and the Prominent Negative Role of Complement Activation on Androgen Synthesis

**Figure 3:**
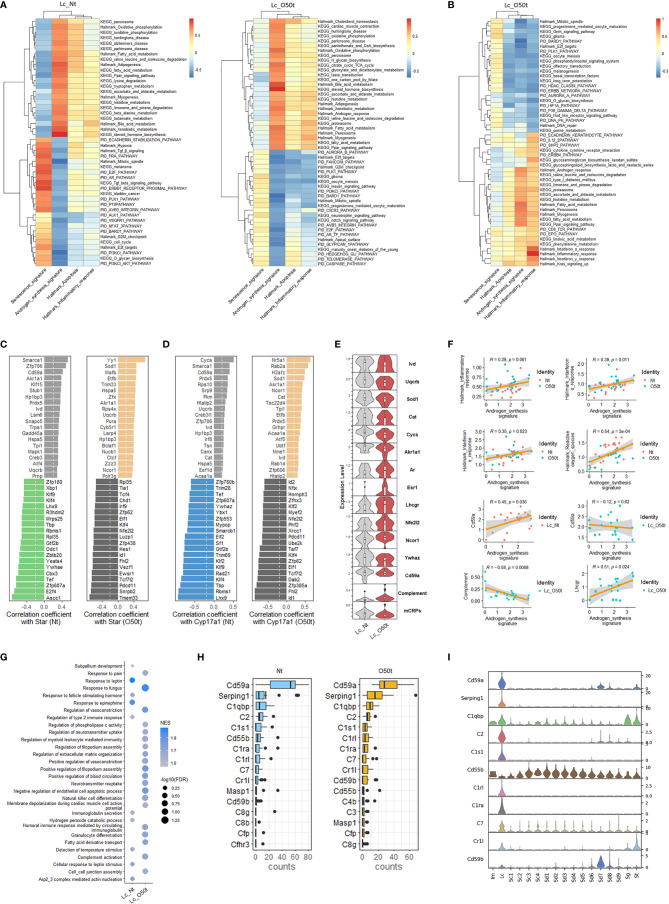
Correlation of androgen synthesis, senescence and inflammation reaction. **(A)** The top correlation ranks of androgen synthesis signature with Hallmark gene sets, KEGG pathways, and PID pathways in Lc_Nt (R ≥ 0.6 and R ≤ −0.45) (left panel) and Lc_O50t (R ≥ 0.6 and R ≤ −0.5) (right panel). **(B)** The top correlation ranks of Hallmark inflammatory response with Hallmark gene sets, KEGG pathways, and PID pathways in Lc_O50t (R ≥ 0.6 and R ≤ −0.5). **(C, D)** The ranks of top 20 positively and negatively correlated TFs with Star (left two panels) or Cyp17a1 (right two panels) in Lc_Nt and Lc_O50t, respectively. **(E)** The expression level of key hormone receptors, mCRPs, complement, and transcription factors positively or negatively correlated with Star or Cyp17a1 in Lc. mCRPs, membrane-bound complement regulatory proteins. **(F)** Correlations of androgen synthesis signature with Hallmark inflammation reaction, Hallmark_interferon-α response, Hallmark_interferon-γ response, Cd59a, and complement and Lhcgr in Lc. **(G)** GSEA analysis (C5-BP) of Lc_Nt and Lc_O50t. The top 10 in Lc_Nt and top 20 in Lc_O50t according to the ranks of −log10(FDR) were displayed. **(H)** The top 16 complements and mCRPs of Lc_Nt and Lc_O50t were shown respectively. **(I)** The overall expression profile of the top 10 complements or mCRPs ranked in Lc_Nt.

Next, we screened signaling pathways closely correlated with androgen synthesis, especially those related to proinflammatory cytokines present during EAO. In Nt and O50t, KEGG oxidative phosphorylation, Hallmark oxidative phosphorylation, KEGG peroxisome, Hallmark peroxisome, KEGG fatty acid metabolism, Hallmark fatty acid metabolism, and KEGG Ppar signaling pathway were highly positively correlated with androgen synthesis, suggesting that normal redox status and fatty acid metabolism pathways were pivotal to androgen synthesis in Lc (**Figure 3A**). Hallmark G2M checkpoint, Hallmark mitotic spindle, Hallmark E2F targets, PID E2F pathway, PID PI3KCI-AKT pathway, and PID PLK1 pathway were negatively correlated with androgen synthesis in Lc_Nt and Lc_O50t, implying that cell cycle insults were negative regulating factors for androgen synthesis. The senescence signature also displayed an opposite correlation pattern from the androgen synthesis signature (**Figure 3A**). Among cytokine pathways, Hallmark interferon-α response and interferon-γ response gene sets displayed the highest correlation with Hallmark inflammatory response (**Figure 3B**). Hallmark interferon-α response, Hallmark interferon-γ response, and Hallmark inflammatory response gene sets also positively correlated with androgen synthesis. Interestingly, KEGG fatty acid metabolism, Hallmark fatty acid metabolism, KEGG Ppar signaling pathway, KEGG peroxisome, Hallmark peroxisome, and Hallmark androgen response, which positively correlated androgen synthesis signature, were also highly correlated with the Hallmark inflammatory response. In addition, Hallmark TGF-β signaling and KEGG TGF-β signaling pathway were negatively correlated with androgen synthesis and positively correlated with senescence in Lc_Nt (**Figure 3A**). PID *Cxcr3* pathway, KEGG insulin signaling pathway, PID caspase pathway, Hallmark G2M checkpoint, and PID Ar_TF pathway, a pathway involved in the regulation of androgen receptor (*Ar*) activity, were negatively correlated with androgen synthesis.

Then, we screened the key transcription factors (TFs) positively or negatively correlated with the rate-limiting enzymes of androgen synthesis to obtain further insight into the unique regulation of androgen synthesis during EAO. Interestingly, the ranks of the top 20 TFs that positively or negatively correlated with the four key androgen synthesis enzymes (*Star*, *Cyp17a1*, *Hsd3b1*, and *Cyp11a1*) changed considerably during EAO, illustrating the possible roles of *Yy1*, *Sod1*, *Cat*, *Nr5a1*, *Akr1a1*, *Ncor1*, and *Hspa5* in supporting androgen synthesis during orchitis (**Figures 3C, D** and **Supplementary Figures S2A, B**). Furthermore, normal androgen synthesis positively correlated with *Hp1bp3*, *Smarca1*, *Cycs*, *Cd59a*, *Zfp706*, *Ivd*, *Uqcrb*, and *Prdx5* in Lc_Nt cells. On the other hand, *Klf2*, *Klf4*, *Nfe2l2/Nrf2*, *Id1*, *Id2*, *Rad21*, *Xrcc1*, and *Cycs* were found to be negatively correlated with androgen synthesis during orchitis. The genes that were reported earlier to negatively regulate androgen synthesis, namely, *Ywhae*, *Ywhaz*, and *Sf1* (36, 37), were also identified in our studies in the top 20 negatively correlated TFs of *Star* or *Cyp17a1.* Our data also revealed that the positive correlation of several TFs with androgen synthesis signature, such as *Nr5a1*, *Yy1*, *Sod1*, and *Ncor1*, was improved during orchitis (**Supplementary Figure S2C**). The level of key enzymes Ivd, Uqcrb, Sod1, Akr1a1, and Cat, which were members of the gene set Hallmark reactive oxygen species, were decreased during orchitis (**Figure 3E**). Their decrease coincided with the decrease in the overall gene sets of Hallmark reactive oxygen species and Hallmark oxidative phosphorylation during orchitis (**Figure 2A**). These key factors may undermine androgen synthesis. Possibly as a compensation, androgen receptor Ar, estrogen receptor Esr1, LH receptor Lhcgr, and Nfe2l2 were all increased during orchitis (**Figure 3E**). This was also evidenced by a positive correlation between inflammation and interferon-α/interferon-γ signaling pathways, and the positive correlation between Lhcgr and androgen synthesis in Lc (**Figure 3F**). To improve the reliability of these correlation analyses, we also performed a correlation analysis of TFs with the four key androgen synthesis enzymes in another mouse testis scRNA-Seq dataset (GSE174731) from the GEO database (**Supplementary Figure S2D**). Both data analyses shared many similar positively correlated TFs with the four androgen synthesis enzymes in normal Lc, such as *Cd59a*, *Smarca1*, *Creb3*, *Creb3l1*, *Ivd*, *Jun*, *Fos*, *Akr1a1*, *Cycs*, *Prdx5*, *cat*, *Prnp*, *Nfia*, *Hp1bp3*, *Pkm*, *Nucb1*, *Irf8*, *Acaa1a*, *Etfb*, *Canx*, and *Tsn*.

More interestingly, Cd59a, which also correlated positively with Star, Cyp17a1, and androgen synthesis in Lc_Nt, was shifted to a weak negative correlation with androgen synthesis during orchitis (**Figures 3C, D, F**). Cd59a was a key member of membrane-bound complement regulatory proteins (mCRPs). We then collected most of the structural components of the complement pathways, designated as a complement signature, and named the gene collection of *Cd59a*, *Cd59b*, *Cd55b*, *Cd55*, and *Cd46* as mCRPs signature to check their correlations with androgen synthesis. As expected, mCRPs were positively correlated with androgen synthesis in Lc_Nt and decreased in Lc_O50t, while complement was negatively correlated with androgen synthesis in Lc_O50t (**Figure 3F**). GSEA pathway enrichment analysis confirmed the prominent expression change of complement during EAO (**Figure 3G**). Many members of complement and mCRPs were expressed prominently in Lc in testis (**Figures 3H, I** **)**.

### Interstitial Fibrosis During EAO and Its Potential Antagonism of Androgen Synthesis

**Figure 4:**
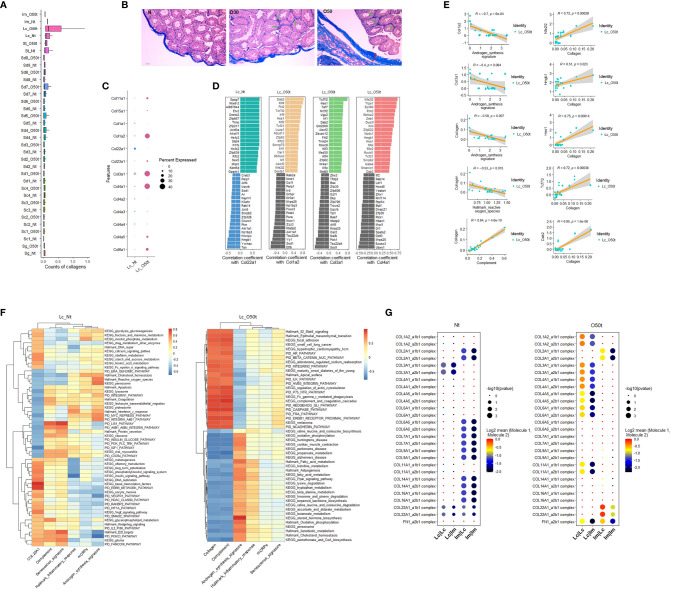
Correlation of testis fibrosis and androgen synthesis during EAO. **(A)** Average expression levels of collagens in each cell cluster of Nt and O50t. **(B)** Masson staining of the fibrosis in the testis slices of Nt and O50t. Tunica albuginea and the interstitial were positive. Green arrows show the stronger fibrosis staining in interstitial spaces of O30 and O50 samples. **(C)** The expression levels of collagen family members in Lc_Nt or Lc_O50t. **(D)** The ranks of top 20 positively and negatively correlated TFs with Col22a1 in Lc_Nt, and Col1a2, Col3a1, and Col4a1 in Lc_O50t. **(E)** Correlations of collagen with androgen synthesis signature, complement, and Hallmark reactive oxygen species and the correlations of five TFs with collagen signature in Lc_O50t. **(F)** The top correlation ranks of Col22a1 (Lc_Nt) or Collagen (Lc_O50t) with Hallmark gene sets, KEGG pathways, and PID pathways in Lc_Nt (R ≥ 0.35 and R ≤ −0.46) (left panel) and Lc_O50t (R ≥ 0.75 and R ≤ −0.57) (right panel). **(G)** The pairing patterns of collagens (receptors) and integrins (ligands) in between Lc vs. Lc, Lc vs. Im, Im vs. Lc, and Im vs. Im in Nt (left) and O50t (right) samples.

To explore the contributing factors of inflammaging during EAO, we also monitored the interstitial fibrosis and the correlated signaling pathways induced by chronic inflammation. In fact, Lc were likely to be one of the main sources of fibrosis because the overall collagen level in Lc was considerably induced during orchitis and was maintained at the highest level among all cell clusters detected in testes (**Figure 4A**). In line with data from earlier reports (28, 38, 39), in addition to the tunica albuginea, interstitial spaces were also positive for fibrosis staining (**Figure 4B**). In all the collagen members detected to be expressed in Lc, the expression profile shifted greatly during EAO, and Col3a1, Col4a1, and Col1a2 became the main collagens expressed during orchitis (**Figure 4C**). By examining the correlated TFs with the three collagen genes in Lc_O50t and Col22a1 in Lc_Nt, we found many TFs highly correlated with the four collagens displayed an opposite pattern with those of Star and Cyp17a1 (**Figure 4D**). The TFs such as *Nfe2l2/Nrf2*, *Hmgb1*, *Hes1*, *Tcf7l2*, *Mettl3*, *Dab2*, and *Ep300* took potentially key roles in positively regulating collagen expression during EAO (**Figures 4D, E** **)**. *Uqcrb*, *Sod1*, *Zfp30*, and *Zzz3* negatively regulated collagen expression during orchitis. The expression of many TFs for collagen expression and androgen synthesis signature displayed a reciprocal relationship. Thus, collagen was revealed to be negatively correlated with androgen synthesis signature and Hallmark reactive oxygen species in Lc_O50t cells (**Figure 4E**).

Then, we used the signature of collagens to screen correlated pathways in Lc_Nt and Lc_O50t. Since the overall expression levels of collagen members in Lc_Nt were low, we used Col22a1 as the signature to screen the correlated pathways in Lc_Nt. Both Col22a1 in Lc_Nt and collagen signature in Lc_O50t shared many similar pathways that negatively correlated with androgen synthesis signature (**Figure 4F**). But during EAO, the highly correlated signaling pathways with collagen showed very high correlation with complement signature and KEGG complement and coagulation cascade pathway. Notably, Hallmark Il2-Stat5 signaling pathway displayed very highly positive correlation with collagen, while KEGG fatty acid metabolism, Hallmark fatty acid metabolism, and many other signaling pathways related to metabolism showed very highly negative correlation with collagen in Lc_O50t. Therefore, cytokines as Il2 were also potentially involved in interstitial fibrosis during orchitis.

More importantly, fibrosis changed the communication and adhesion patterns among Lc and Im cells. For example, the number of receptor–ligand pairs of collagen-integrin in Im vs. Lc, and Im vs. Im were decreased in Lc_O50t (**Figure 4G**). However, the number of receptor–ligand pairs of collagen-integrin in Lc vs. Lc and Lc vs. Im was increased during orchitis. The information suggested that fibrosis during orchitis changed the communication pattern between Lc and macrophages.

In summary, fibrosis and complement were closely correlated, and both potentially antagonized androgen synthesis and enhanced Lc senescence during EAO. The downregulation of Hallmark reactive oxygen species and upregulation of complement pathway were closely related to interstitial fibrosis.

### Most Proinflammatory Cytokines Secreted During EAO Enhanced Apoptosis and Senescence and Antagonized the Glutathione Metabolism Pathway in Lc and Sg

**Figure 5:**
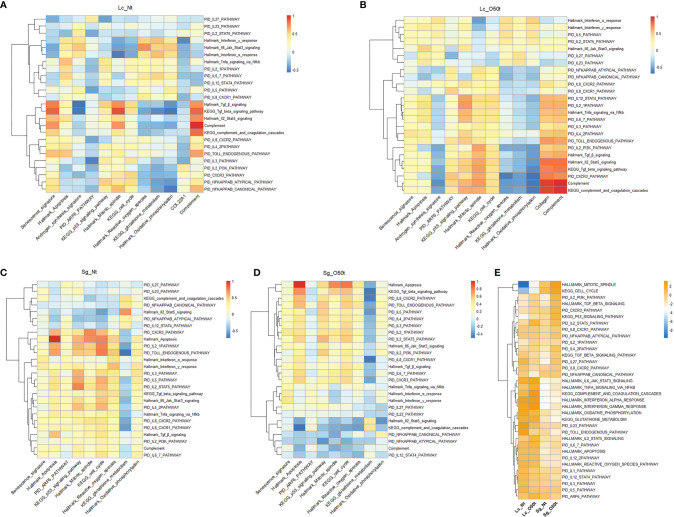
Distinct responses of Lc and Sg to proinflammatory factors during EAO. **(A–D)** Correlation of selected signatures or pathways with 25 pathways of proinflammatory factors in Lc_Nt, Lc_O50t, Sg_Nt, and Sg_O50t, respectively. **(E)** The relative expression levels of the associated pathways in **(A–D)** in Lc_Nt, Lc_O50t, Sg_Nt, and Sg_O50t.

Proinflammatory cytokines and chemokines are known to be the main regulatory factors that contribute to apoptosis of testicular cells during inflammation (24, 40–46). In this study, we examined for any correlations between 25 proinflammatory cytokine gene sets, signaling pathways, and signatures in Lc and Sg. In Lc_O50t, most cytokine signaling pathways were positively correlated with both senescence signature and Hallmark apoptosis while negatively correlated with androgen synthesis signature, suggesting that most proinflammatory cytokines potentially enhanced both the senescence and apoptosis and decreased androgen production in Lc during EAO (**Figures 5A, B** **)**. In Sg_Nt, most cytokines were weakly negatively correlated with senescence signature and positively correlated with Hallmark apoptosis (**Figure 5C**). In contrast, in Sg_O50t, most cytokines were positively correlated with both senescence and Hallmark apoptosis (**Figure 5D**). Therefore, proinflammatory cytokines during orchitis induced both senescence and apoptosis in Lc and Sg, which is in line with the reported Sg apoptosis during EAO induced by LPS or testicular homogenate with adjuvants (46–48).

To further elucidate the underlying mechanism, we checked the highly positive signaling pathways related to senescence and apoptosis for Lc and Sg. PID Arf6 pathway and KEGG p53 signaling pathway, two typical senescence-related pathways, were shown to be correlated with senescence signature both in Lc_O50t and Sg_O50t (**Figure 2E** and **Supplementary Figure S3A**). Hallmark mitotic spindle and KEGG cell cycle were positively correlated with Hallmark apoptosis in Sg_O50t (**Figure S3B**). Therefore, these signaling pathways were selected to monitor their correlation with the 25 proinflammatory cytokine-related signaling pathways and signatures. In Lc_O50t, most cytokines were positively correlated with the PID Arf6 pathway and KEGG p53 signaling pathway, suggesting that both signaling pathways were related to the senescence induced by proinflammatory cytokines in Lc during orchitis (**Figure 5B**). At the same time, these cytokine pathways positively correlated with Lc senescence were also positively correlated with Hallmark apoptosis in Lc_O50t (**Figure 5B**). In Sg cells, most cytokine pathways were shown to be positively correlated with PID Arf6 pathway in Sg_Nt and shifted to negative and low correlation with PID Arf6 pathway in Sg_O50t (**Figure 5D**). However, most cytokine signaling pathways kept a positive correlation with Hallmark mitotic spindle and KEGG cell cycle, the two apoptosis-related signaling pathways, in both Sg_Nt and Sg_O50t (**Figures 5C, D** **)**. The data revealed that proinflammatory cytokines were inclined to induce both senescence and apoptosis during orchitis in both Lc and Sg, but the Arf6 pathway displayed a different response towards these cytokines in Lc and Sg. The KEGG p53 signaling pathway, Hallmark mitotic spindle, and KEGG cell cycle seemed to be important in mediating most cytokine signaling pathways-induced senescence and apoptosis both in Lc and Sg during orchitis (**Figures 5B, D** **)**.

Then, we screened the negative correlated pathways with most proinflammatory cytokines both in Lc and Sg during EAO. Nine pathways were screened, all of which were derived from metabolism pathways (**Supplementary Figures S3C, D**). Among the nine pathways, KEGG_glutathione_metabolism and KEGG_metabolism_of_xenobiotics_by_cytochrome_p450 showed the most negative correlation with most proinflammatory cytokine pathways during EAO, suggesting that redox metabolism were the most important target by proinflammatory cytokines. Therefore, KEGG_glutathione_metabolism was picked for further correlation analysis. Most proinflammatory cytokines enhanced fibrosis and complement in Lc_O50t and at the same time decreased Hallmark oxidative phosphorylation, Hallmark reactive oxygen species, and KEGG glutathione metabolism (**Figure 5B**). These redox-related pathways were extremely pivotal for maintaining the normal redox status and clearing the reactive species generated during androgen synthesis in Lc. In Sg_O50t, most proinflammatory cytokines enhanced Hallmark oxidative phosphorylation and Hallmark reactive oxygen species but still antagonized KEGG glutathione metabolism (**Figure 5D**). Therefore, compromised glutathione metabolism was potentially a common insult both for Lc and Sg cells during chronic EAO and thus became a potential pathological target of proinflammatory cytokines in orchitis. This was also evidenced by the fact that TNF-α inhibits glutathione S-transferase-α in Sertoli cells (49). Notably, the expression levels of most cytokine/chemokine pathways and redox-related pathways were higher in Lc than those in Sg, while the levels of cell-cycle-related pathways and p53 signaling pathway were much higher in Sg (**Figure 5E**), which may account for the distinct responses of Lc and Sg to proinflammatory cytokines/chemokines. Overall, the pathway involved in glutathione metabolism was the key potential target by most proinflammatory cytokines/chemokines derived from the correlation analysis of pathways both in Lc and Sg.

## Discussion

Using high throughput data analysis, screening for correlations between genes and disease phenotype can provide pivotal insights into deciphering pathological disease mechanisms. During high throughput data analysis, the chosen representative signature of disease phenotype is key. For example, gene collections representative of senescence for both young and aging animals is important in senescence evaluation (**Figures 2D–F**).

A limitation is differentially expressed genes either with an up- or downregulation pattern may not reflect changes in whole pathways or gene networks during certain pathological changes. Gene differential analysis through the analysis of changes in the expression of target genes over a defined cutoff also leads to considerable loss of valuable information. More valuable information can be gathered by evaluating the relative change in expression profile in the scale of an entire signaling pathway or a whole gene set. Therefore, GSEA was proposed many years ago and became a routine and popular tool for data mining (50, 51). Differentially expressed genes or GSEA analysis, however, still does not represent the antagonistic or synergistic relationship of these genes and gene sets with disease phenotype. Correlation can be categorized as positive correlation, negative correlation, and no correlation. Proinflammatory cytokines in EAO changed the correlation of many gene sets or signaling pathways with androgen synthesis, senescence, and apoptosis. Such information was usually hard to be obtained from differentially expressed genes and thus was easily missed during data mining. For example, KEGG glutathione metabolism was revealed to be a key target of many proinflammatory cytokines by correlation analysis during EAO (**Figures 5A–D**), but its expression in Lc and Sg between Nt and O50t was not visibly changed in our EAO model (**Figure 5E**).

Correlation analysis can provide an alternate method to evaluate for positively or negatively correlated genes with certain key genes or valuable signatures of disease phenotypes. In this study, we identified a plethora of related TFs to rate-limiting enzymes in androgen synthesis (**Figures 3C, D** and **Supplementary Figures S2A, B, D**). We also identified some key TFs potentially contributing to fibrosis, such as Nfe2l2, Hmgb1, Hes1, Tcf7l2, and Dab2 (**Figures 4D, E** **)**. *Nfe2l2/Nrf2* is the master regulator of antioxidative responses and plays a prominent role in protecting against inflammation under normal physiological conditions (52, 53). In chronic pathological conditions, however, Nfe2l2/Nrf2 can be a stimulator of fibrosis. For example, *Nfe2l2/Nrf2* enhances fibrosis during the chronic stage of alcoholic liver disease (54, 55), which may explain the surprising positive correlation of *Nfe2l2/Nrf2* with fibrosis in this study (**Figures 4D, E** **)**. This example of *Nfe2l2* was attributed to the use of correlation analysis.

One thing that should be noted is that senescence, fibrosis, complement, and a plethora of proinflammatory cytokines can lead to compromised androgen synthesis. However, many other inflammatory factors also improve androgen synthesis during orchitis. For example, Hallmark inflammatory response pathway itself and some cytokine pathways such as the PID Il5 pathway, Hallmark interferon-α response, and interferon-γ response pathways also displayed slight improvement on androgen synthesis (**Figures 3F**, **5A, B**). Additionally, the sex hormone receptors Ar, Esr1, and Lhcgr were also increased in Lc_O50t (**Figure 3E**) and distinct to aging testes (56, 57). The regulation of androgen synthesis was thus influenced by the interactions and balances of multiple antagonizing and synergistic signaling pathways. Different orchitis models, different inflammatory insults, and distinct inflammatory phases may result in variable serum testosterone level in testes (21, 58–62). When germ cells undergo enormous apoptosis and trigger Lc proliferation *via* endocrine feedback, more androgen secretion by Lc occurs in EAO (58). Upon such variable cases, the use of correlation analysis is thus feasible to screen and dissect the targeted variable signaling pathways with certain signature.

The feasibility of this analysis in screening androgen synthesis-related pathways is also confirmed by the well-known pathways reported earlier (3). Furthermore, some of the screened genes positively correlated with androgen synthesis have been verified by experiments, such as *Yy1* (63), *Nr5a1/Sf-1* (64), *Hspa5/Grp78* (60), *Clock* (65), *Tsn/*Translin (60), and *Fos* and *Jun* (60). Therefore, correlation screening is a useful tool for scRNA-seq data mining.

Another pivotal study finding worth highlighting is that correlation analysis identified an antagonizing relationship between most proinflammatory cytokines and chemokines with the glutathione metabolism pathway. Glutathione is the master antioxidant in cells, which can facilitate maintaining a normal environment upon oxidative stress by various inflammatory cytokines and chemokines. Our theory in this study thus supports the potential use of antioxidative therapy in chronic inflammation and severe infections. Interestingly, glutathione has been recommended to be an adjunctive reagent in the treatment of severe coronavirus disease 2019 (COVID-19) patients (66–69).

Taken collectively, correlation coefficient analysis of various signaling pathways and genes can further elucidate gene regulation networks and their subsequent changes during stress, and this is a distinct advantage of utilizing large sample data such as scRNA-Seq dataset(s) for data mining. In this study, we identified many novel signaling pathways and genes correlating with signatures of androgen synthesis, senescence, or fibrosis. Although these correlations need further verification in future investigations, our findings provide new important insights regarding the regulation of androgen synthesis and the knowledge of inflammatory senescence in Leydig cells in orchitis.

## Data Availability Statement

The datasets presented in this study can be found in online repositories. The names of the repository/repositories and accession number(s) can be found below: https://www.ncbi.nlm.nih.gov/geo, GSM5563668 and GSM5563669.

## Ethics Statement

The animal study was reviewed and approved by University of Nantong Animal Care and Use Committee and the Animal Care and Use Office.

## Author Contributions

FS, CC, and YT performed supervision, funding acquisition, project administration, and methodology. PM performed most experiments presented in the manuscript: cell isolation, HE, IF, and Masson staining. JW performed animal handling, cell preparation, and data curation. WQ and XL provided technical assistance, project administration, and supervision. JC and YH performed animal model preparation. YL performed data analysis and experimental design and wrote the manuscript. All authors contributed to the article and approved the submitted version.

## Funding

This work was supported by the National Key Research and Development Program of China (No. 2018YFC1003500 to FS), the open project of NHC Key Laboratory of Male Reproduction and Genetics of China (No. KF201806 to YL), and the National Natural Science Foundation of China (No. 31271448 to YL).

## Conflict of Interest

The authors declare that the research was conducted in the absence of any commercial or financial relationships that could be construed as a potential conflict of interest.

## Publisher’s Note

All claims expressed in this article are solely those of the authors and do not necessarily represent those of their affiliated organizations, or those of the publisher, the editors and the reviewers. Any product that may be evaluated in this article, or claim that may be made by its manufacturer, is not guaranteed or endorsed by the publisher.
