# Supplementary figures and images for: High Throughput scRNA-Seq Provides Insights Into Leydig Cell Senescence Induced by Experimental Autoimmune Orchitis: A Prominent Role of Interstitial Fibrosis and Complement Activation
Source: Front Immunol. 2022 Jan 17;12:771373. doi: 10.3389/fimmu.2021.771373 (PMC8801941; doi:10.3389/fimmu.2021.771373)

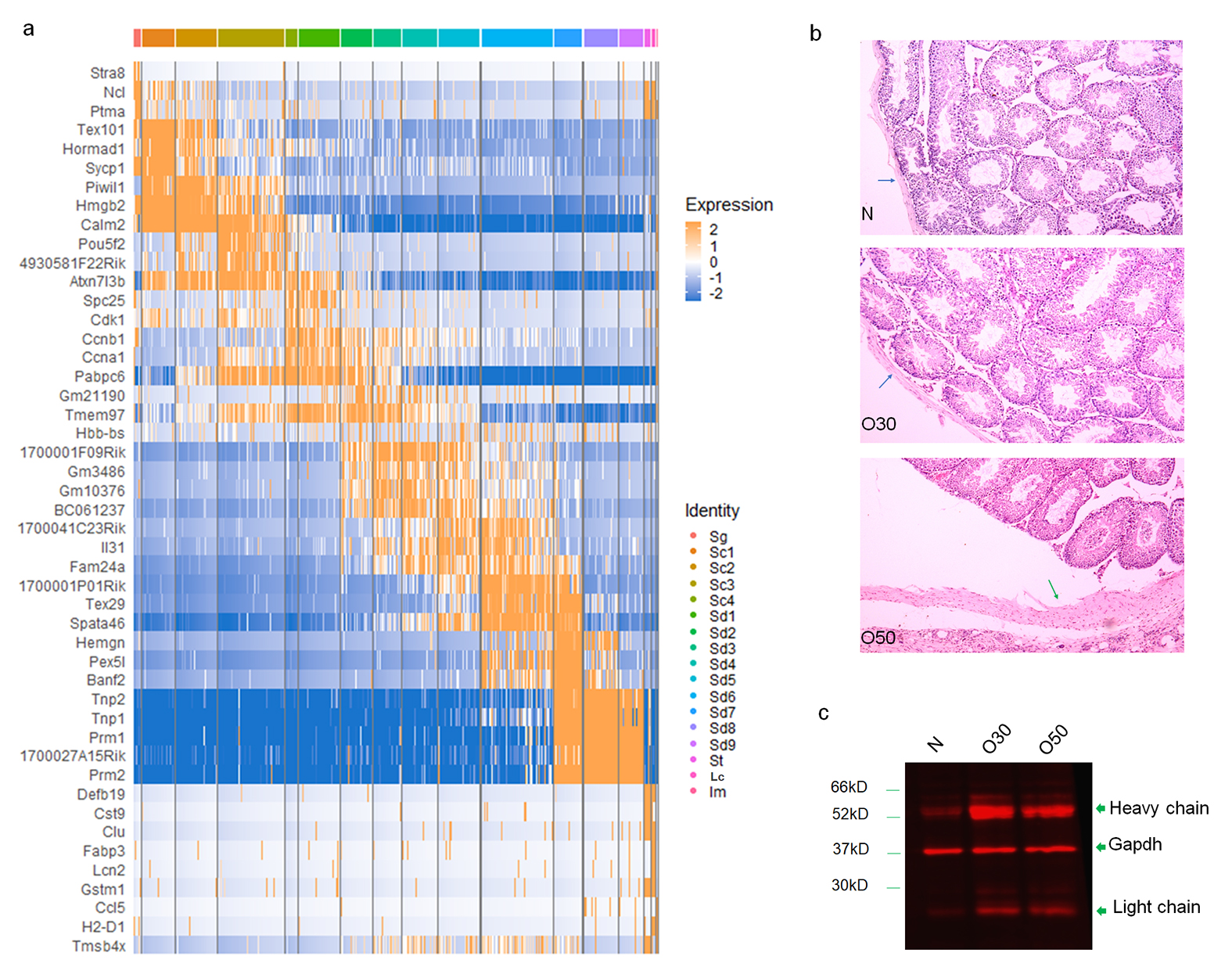

Supplement: Supplementary file 1 [file Figure_S1.jpg]

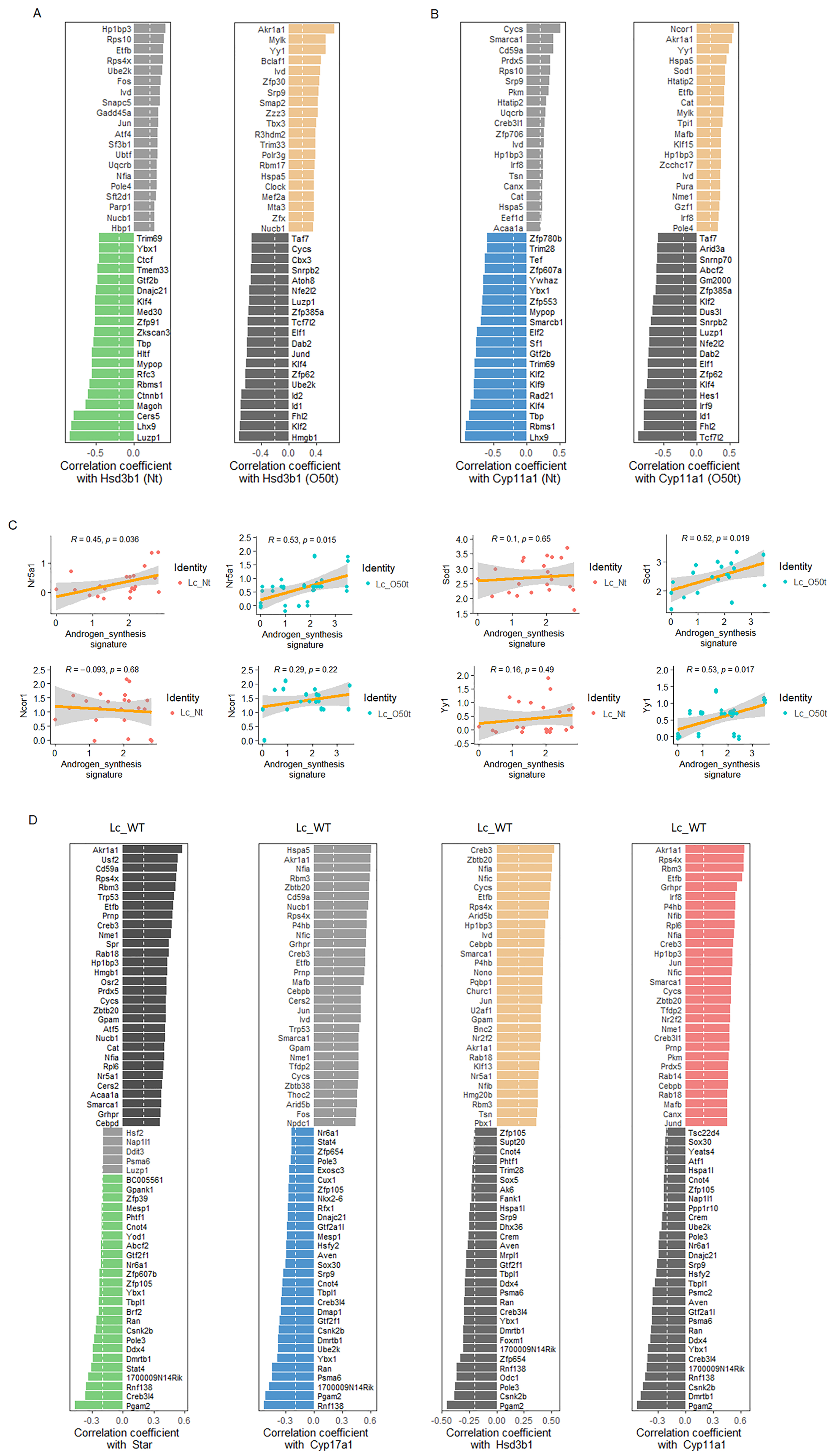

Supplement: Supplementary file 2 [file Figure_S2.jpg]

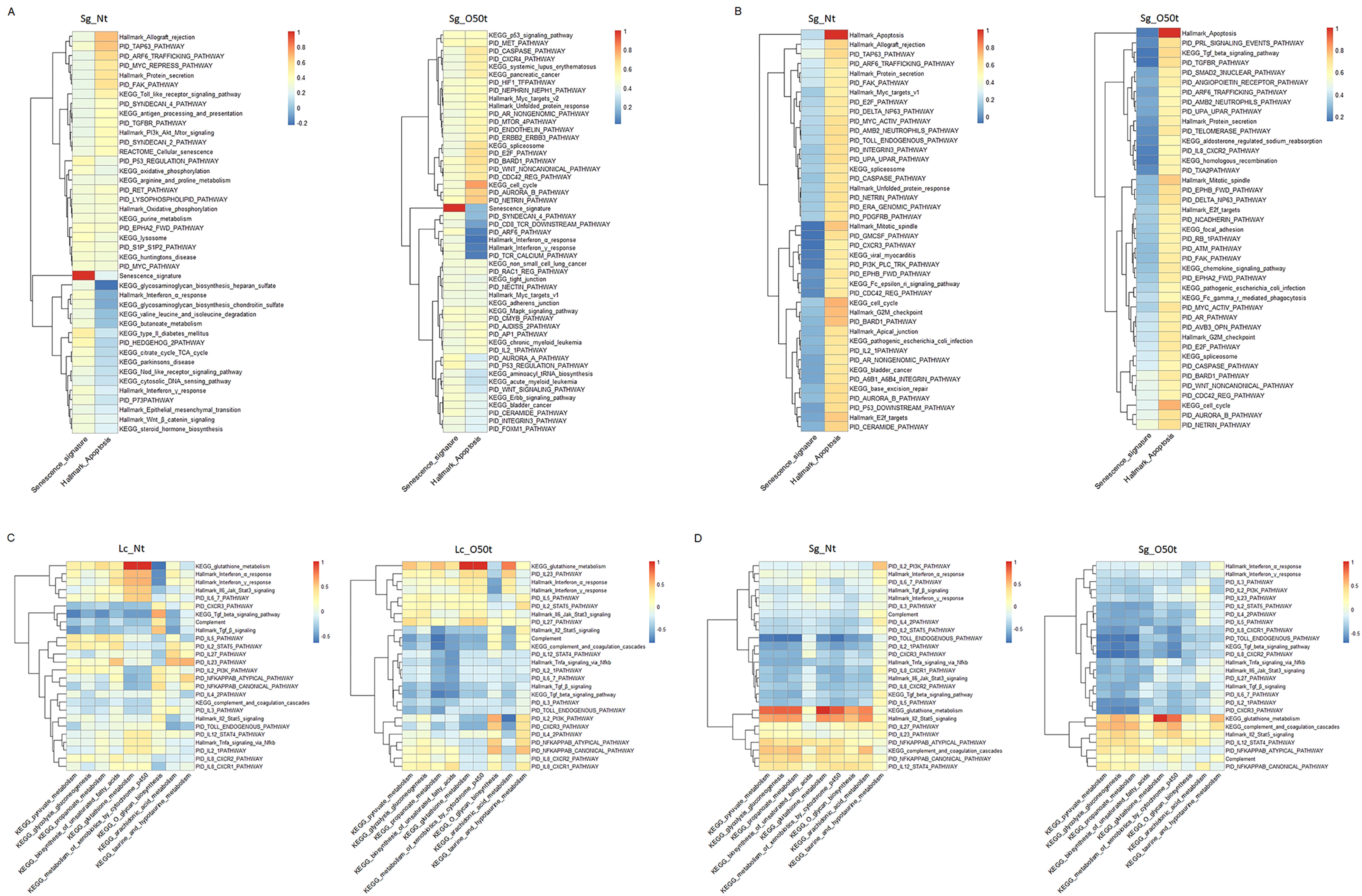

Supplement: Supplementary file 3 [file Figure_S3.jpg]
