# Supplementary material for: High Throughput scRNA-Seq Provides Insights Into Leydig Cell Senescence Induced by Experimental Autoimmune Orchitis: A Prominent Role of Interstitial Fibrosis and Complement Activation
Source: Front Immunol. 2022 Jan 17;12:771373. doi: 10.3389/fimmu.2021.771373 (PMC8801941; doi:10.3389/fimmu.2021.771373)
Supplement: Supplementary file 4 [file Table_S1.pdf]

Signatures	Genes
Senescence_signature	"Cdkn1a","Cdkn1b","Cdkn1c","Cdkn2a","Cdkn2b","Cdkn2c","Cdkn2d"
Androgen_synthesis_signature	"Cyp11a1","Cyp17a1","Hsd3b1","Star"
mCRPs	Cd59a', 'Cd59b', 'Cd55b', 'Cd55', 'Cd46'
Complement	Serping1', 'C1qbp', 'C2', 'C1s1', 'C1ra', 'C1rl', 'C7', 'Cr1l', 'C4b', 'C3', 'Masp1', 'Cfp', 'C8g', 'C8b', 'C1qb', 'Cfhr3', 'C6', 'C1qc', 'C1qa', 'C3ar1', 'Cfh', 'Colec11', 'Hc', 'C4a', 'Cfhr1', 'C8a', 'C9', 'A1cf', 'Crp', 'C1ql4', 'C1s2', 'Mbl2', 'C4bp', 'Masp2', 'C1rb', 'Cr2', 'C1ql1', 'Cfi', 'Cfb', 'Cr1l'
Collagen	Col11a1', 'Col11a2', 'Col12a1', 'Col13a1', 'Col14a1', 'Col15a1', 'Col16a1', 'Col17a1', 'Col18a1', 'Col19a1', 'Col1a1', 'Col1a2', 'Col22a1', 'Col23a1', 'Col26a1', 'Col27a1', 'Col28a1', 'Col2a1', 'Col3a1', 'Col4a1', 'Col4a2', 'Col4a3', 'Col4a4', 'Col4a5', 'Col4a6', 'Col5a1', 'Col5a2', 'Col6a1', 'Col6a2', 'Col6a3', 'Col7a1', 'Col8a1', 'Col9a1', 'Col9a3'
